# Draft Genome Sequences of *Escherichia coli* Isolates from Wounded Military Personnel

**DOI:** 10.1128/genomeA.00828-16

**Published:** 2016-08-11

**Authors:** Brock A. Arivett, Dave C. Ream, Steven E. Fiester, Destaalem Kidane, Luis A. Actis

**Affiliations:** a Department of Microbiology, Miami University, Oxford, Ohio, USA; b Biology Department, Middle Tennessee State University, Murfreesboro, Tennessee, USA

## Abstract

Members of the *Escherichia coli* bacterial family have been grouped as ESKAPE (*Enterococcus faecium*, *Staphylococcus aureus*, *Klebsiella pneumoniae*, *Acinetobacter baumannii*, *Pseudomonas aeruginosa*, and *Enterobacter* species) pathogens because of their extensive drug resistance phenotypes and increasing threat to human health. The genomes of six extended-spectrum β-lactamase (ESBL)-producing *E. coli* strains isolated from wounded military personnel were sequenced and annotated.

## GENOME ANNOUNCEMENT

*Escherichia coli* is a Gram-negative bacillus that is a component of the normal bacterial flora of the human colonic tract (1–3). *E. coli* within the intestinal tract is typically nonpathogenic unless the intestinal tract barriers have been violated or the host has been immunocompromised; however, there are also several highly adapted *E. coli* clones that have evolved to cause human diseases including meningitis, diarrhea, sepsis, and urinary tract infections (3). In fact, *E. coli* is the most common Gram-negative pathogen associated with nosocomial infections, and isolates with the ability to produce extended-spectrum β-lactamase (ESBL) continue to increase in frequency and severity (4, 5). The genomes of six ESBL-producing isolates obtained from wounded soldiers at the Walter Reed Army Medical Center (WRAMC) were sequenced using next-generation sequencing methods for future analyses to elucidate their virulence mechanisms.

As described previously, strains were routinely stored at −80°C in 10% glycerol (6). DNA was isolated from overnight LB cultures grown with agitation at 37°C using the DNeasy blood and tissue kit (Qiagen, Valencia, CA, USA). Absorption at 260 nm and 280 nm was measured for each sample to determine DNA quantity and quality using a Nanodrop 2000 (Thermo Scientific, Wilmington, DE, USA). DNA concentrations for library preparation were determined by the SYBR green (Life Technologies, Grand Island, NY, USA) standard curve method in black 96-well plates (Corning, Tewksbury, MA, USA) using a FilterMax F5 spectrophotometer with Multi-Mode Analysis software version 3.4.0.25 (Molecular Devices, Sunnyvale, CA, USA). Whole DNA was sheared to approximately 500 bp in a microTUBE-50 using the M220 Focused-ultrasonicator (Covaris, Woburn, MA, USA). Fragmentation of resultant libraries was examined with a Bioanalyzer 2100 high sensitivity DNA analysis kit (Agilent Technologies, Santa Clara, CA, USA) using version B.02.08.SI648 software. Individual libraries were normalized, pooled, and then sequenced using MiSeq v3 600-cycle kit (Illumina, San Diego, CA, USA) to perform 300-bp paired-end sequencing on a MiSeq instrument (Illumina) per manufacturer’s instructions. *De novo* assembly was performed using Genomics Workbench 8.0 with the bacterial genome finishing module (CLC bio, Boston, MA, USA) on a workstation with an AMD Opteron 2.10 GHz 16-core processor with 128 GB DDR3 ECC RAM. Prokka version 1.10 on a quadcore i7 workstation with 32 GB DDR3 running Ubuntu 14.04 LTS (7) was used for genome annotation. The *de novo* assembly statistics for the sequenced *E. coli* isolates are shown in Table 1.

**TABLE 1:** Assembly metrics and accession numbers of *Escherichia coli* genomes

Strain ID	No. of contigs	*N*_50_ contigs (bp)	Total size (bp)	Coverage (×)	G+C content (%)	No. of ORFs^a^	No. of RNAs	Accession no.
105454	101	191,011	5,335,253	16	50.68	5,121	70	LOJL00000000
105547	93	127,705	4,858,207	18	50.68	4,649	74	LOJM00000000
109497	99	222,697	5,221,557	34	50.78	5,050	74	LORD00000000
108191	98	115,521	4,727,016	28	50.81	4,518	73	LORE00000000
105433	89	138,092	4,820,700	33	50.74	4,556	73	LORF00000000
105438	81	204,373	5,226,940	34	50.68	4,996	60	LORC00000000

a Open reading frames.

### Accession number(s).

The whole-genome shotgun projects were deposited into GenBank under Bioproject ID PRJNA261239 with the accession numbers listed in Table 1.
